# Co‐ordination of the upper and lower limbs for vestibular control of balance

**DOI:** 10.1113/JP274272

**Published:** 2017-09-22

**Authors:** Craig P. Smith, Jonathan E. Allsop, Michael Mistry, Raymond F. Reynolds

**Affiliations:** ^1^ School of Sport, Exercise and Rehabilitation Sciences University of Birmingham Birmingham UK; ^2^ Vision and Eye Research Unit, Postgraduate Medical Institute Anglia Ruskin University Cambridge UK; ^3^ School of Informatics University of Edinburgh Edinburgh UK

**Keywords:** Upper limb, Vestibular system, Balance, Galvanic vestibular stimulation

## Abstract

**Key points:**

- When standing and holding an earth‐fixed object, galvanic vestibular stimulation (GVS) can evoke upper limb responses to maintain balance.
- In the present study, we determined how these responses are affected by grip context (no contact, light grip and firm grip), as well as how they are co‐ordinated with the lower limbs to maintain balance.
- When GVS was applied during firm grip, hand and ground reaction forces were generated.
- The directions of these force vectors were co‐ordinated such that the overall body sway response was always aligned with the inter‐aural axis (i.e. craniocentric).
- When GVS was applied during light grip (< 1 N), hand forces were secondary to body movement, suggesting that the arm performed a mostly passive role.
- These results demonstrate that a minimum level of grip is required before the upper limb becomes active in balance control and also that the upper and lower limbs co‐ordinate for an appropriate whole‐body sway response.

**Abstract:**

Vestibular stimulation can evoke responses in the arm when it is used for balance. In the present study, we determined how these responses are affected by grip context, as well as how they are co‐ordinated with the rest of the body. Galvanic vestibular stimulation (GVS) was used to evoke balance responses under three conditions of manual contact with an earth‐fixed object: no contact, light grip (< 1 N) (LG) and firm grip (FG). As grip progressed along this continuum, we observed an increase in GVS‐evoked hand force, with a simultaneous reduction in ground reaction force (GRF) through the feet. During LG, hand force was secondary to the GVS‐evoked body sway response, indicating that the arm performed a mostly passive role. By contrast, during FG, the arm became actively involved in driving body sway, as revealed by an early force impulse in the opposite direction to that seen in LG. We then examined how the direction of this active hand vector was co‐ordinated with the lower limbs. Consistent with previous findings on sway anisotropy, FG skewed the direction of the GVS‐evoked GRF vector towards the axis of baseline postural instability. However, this was effectively cancelled by the hand force vector, such that the whole‐body sway response remained aligned with the inter‐aural axis, maintaining the craniocentric principle. These results show that a minimum level of grip is necessary before the upper limb plays an active role in vestibular‐evoked balance responses. Furthermore, they demonstrate that upper and lower‐limb forces are co‐ordinated to produce an appropriate whole‐body sway response.

## Key points

- When standing and holding an earth‐fixed object, galvanic vestibular stimulation (GVS) can evoke upper limb responses to maintain balance.
- In the present study, we determined how these responses are affected by grip context (no contact, light grip and firm grip), as well as how they are co‐ordinated with the lower limbs to maintain balance.
- When GVS was applied during firm grip, hand and ground reaction forces were generated.
- The directions of these force vectors were co‐ordinated such that the overall body sway response was always aligned with the inter‐aural axis (i.e. craniocentric).
- When GVS was applied during light grip (< 1 N), hand forces were secondary to body movement, suggesting that the arm performed a mostly passive role.
- These results demonstrate that a minimum level of grip is required before the upper limb becomes active in balance control and also that the upper and lower limbs co‐ordinate for an appropriate whole‐body sway response.

**Abbreviations**
AD: angular deviation
FG: firm grip
LG: light grip
GRF: ground reaction force
GVS: galvanic vestibular stimulation
NC: no contact

## Introduction

Holding onto a solid object improves standing balance. This can be a result of improved sensory information and/or mechanical support, depending upon the nature of the manual contact. For example, light touch with an earth‐fixed object can reduce sway even when forces are too low to offer significant mechanical support (< 1 N) (Jeka & Lackner, 1994; Kouzaki & Masani, 2008). This has also been shown for light touch with another standing person (Reynolds & Osler, 2014). In both cases, the upper limb provides proprioceptive feedback of body sway. Firmer grip can additionally provide mechanical support in the case of a loss of balance, exerting larger forces through the hand to keep the body upright (Maki & McIlroy, 2006). Hence, the arm plays a dual role for balance, as both sensor and motor.

Upper limb motor output for balance has previously been demonstrated using vestibular perturbations. For example, galvanic vestibular stimulation (GVS) has been shown to evoke upper limb responses when forced to use the arm for balance (Britton *et al*. 1993). GVS involves small electrical currents passed across the skin between the mastoid processes. This modulates the activity of the vestibular nerve, producing a false sensation of body position from vertical towards the cathodal electrode when standing (Fitzpatrick & Day, 2004; Reynolds & Osler, 2012). This, in turn, evokes a compensatory body movement response towards the anode electrode. Britton *et al*. (1993) used this stimulus to evoke triceps muscle responses in standing subjects who were firmly grasping a handrail. These responses were only observed in the arm that was actively engaged in the balance task. However, subjects stood on a freely rotating pivot which prevented them from generating ankle torque. Hence, they were forced to use the hand to maintain balance. Whether such responses would be seen during normal stance remains open to question. Furthermore, whether the response would be altered by changes in hand grip is unknown. During light grip (LG) (< 1 N), the arm acts mainly as a sensory organ (Jeka & Lackner, 1994), which suggests that a firmer grip may be required to generate active responses to a vestibular perturbation.

Another aspect of the GVS‐evoked balance response is its dependence on head orientation. When standing normally, the whole‐body sway response to GVS is always directed towards the anodal ear. If the head is turned, the direction of the evoked sway response turns by an equal amount. This ‘craniocentric’ behaviour demonstrates the conversion of vestibular information from a head‐ to body‐centred reference frame. Craniocentric sway responses to GVS have been demonstrated for whole‐body sway and ground reaction forces (GRF) when standing unsupported (Lund & Broberg, 1983; Pastor *et al*. 1993; Mian & Day, 2009; Reynolds, 2011). However, the hand force vector evoked by GVS when holding a fixed object has not been studied. Recent evidence suggests that the direction of GVS responses may not behave in a simple craniocentric fashion. Mian & Day (2014) reported that the direction of the evoked GRF vector is biased towards the direction of least postural stability. For example, touching an earth‐fixed object directly to the right preferentially stabilized baseline sway in the mediolateral axis. Under these circumstances, the GVS response direction became biased towards the anteroposterior axis. Such deviations from the craniocentric principle may also apply to the upper limb force vector.

In the present study, we addressed these issues by studying force responses evoked by GVS in the upper limb when holding onto a fixed object. We aimed to investigate: (i) whether the magnitude and direction of GVS‐evoked upper limb force depends upon grip context; (ii) whether the direction of this force vector is systematically altered by head orientation in a craniocentric fashion; and (iii) how well upper limb force is integrated with the GRF vector, as well as how this affects whole‐body sway. To answer these questions, we asked volunteers to adopt different grip strengths and head orientations when we measured force and body sway responses to GVS.

## Methods

### Ethical approval

Ethical approval was obtained from the University of Birmingham Ethics Committee and was in compliance with the *Declaration of Helsinki*. Informed written consent was obtained from all participants.

### Subjects

Ten subjects completed Experiment 1 (27.2 ± 5.2 years; seven males, three females) and twelve subjects completed Experiment 2 (27.3 ± 6.7 years; 10 males, two females). Subjects were healthy, with no known history of vestibular or neurological disorders.

### Apparatus

**Figure 1:**
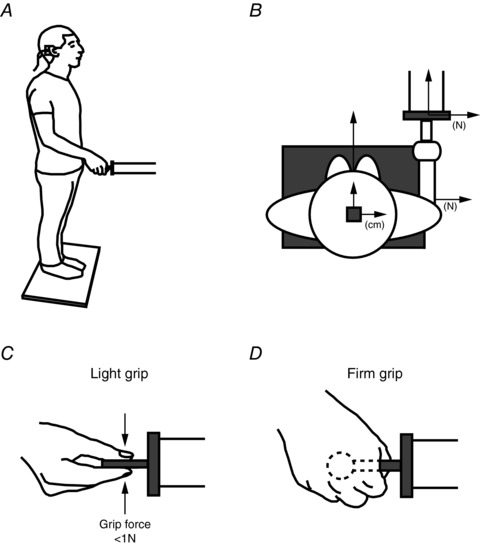
Experimental set‐up *A*, subjects stood barefoot on a force plate with eyes closed, grasping a fixed support. GVS was applied via electrodes placed over the mastoid processes. *B*, set‐up from above. The end effector of the support was positioned forward/right 45° to the subject. Hand force was measured by a force sensor embedded in the support. Head‐on‐body orientation and whole‐body movement were derived by a motion capture sensor positioned on top of the head. *C*, LG (from above): thumb and forefinger gently grasp a grip force sensor < 1 N. *D*, FG (from above): a sphere is firmly grasped in the palm of the hand.

The experimental set‐up is illustrated in Fig. 1. Subjects stood barefoot with feet together on a force plate (Kistler 9286AA; Kistler Instrumente AG, Winterhur, ZH, Swizerland). The end effector of an earth‐fixed support with an embedded triaxial force sensor (HapticMaster; Moog FCS, Nieuw‐Vennep, NH, The Netherlands) was positioned forward/right (35 cm forward of the ankle, 35 cm right of body mid‐line) 45° of the subject, at a height of 110 cm. A motion tracking sensor was used to record sway and head orientation (Fastrak; Polhemus Inc., Colchester, VT, USA) and was attached to the top of a welding helmet frame worn by the subject. All signals were recorded at 100 Hz. Note that forces always refer to forces acting on the body. Fastrak Euler angles were used to derive head yaw (Reynolds, 2011). GVS stimuli were delivered by an isolated constant‐current stimulator (Model 2200; A‐M Systems, Sequim, WA, USA) to gel‐coated carbon rubber electrodes (46 × 37 mm) placed over the mastoid processes in a binaural bipolar configuration.

### General protocol

Each trial consisted of 15 s of quiet standing before a series of 20 GVS stimuli (2 mA of 2 s in duration) was delivered, with a gap of 5 s between each stimulus. Equal numbers of anode‐right and left stimuli were delivered in a random order.

To measure GVS‐evoked responses, signals were aligned to the time point of GVS onset and averaged for each condition. Responses to anode‐left and right currents were found to be equal and opposite (see Results, Experiment 1). Therefore, for all further analysis, both polarities were combined after inverting anode‐left data.

### Experiment 1

In Experiment 1, we determined how the GVS‐evoked upper limb response is altered by changes in grip.

Subjects either stood freely (no contact), lightly grasping the support with thumb and forefinger (LG) (Fig. 1 *C*) or firmly grasping the support with their right hand (firm grip, FG) (Fig. 1 *D*). In LG conditions, a force sensor (50 × 50 × 8 mm; F306 Disc Loadcell; Novatech Measurements Ltd, Hastings, UK) was used as the end effector, allowing measurement of grip force. Subjects were instructed to lightly grip the effector with their right thumb and forefinger. Before data recording, they were shown real‐time feedback of the force signal, which allowed them to practice maintaining grip < 1 N for the LG condition. In the FG condition, a solid sphere (diameter 40 mm) was used as the effector. Subjects were instructed to firmly grip the sphere in the palm of their right hand. In the no contact (NC) conditions, the arms were positioned in front of the subject with hands clasped together. The head was always facing forward and eyes were closed throughout. A trial (15 s of quiet standing before a series of 20 GVS stimuli) was repeated twice for each of the three grip conditions (NC, LG and FG).

The GVS‐evoked ground reaction force (GRF) response consists of a small early component directed towards the cathodal ear (∼250 ms post‐stimulus onset) and a much larger late component directed towards the anodal ear (∼450 ms). The late component is responsible for producing whole‐body movement in compensation for a sense of self‐motion (Marsden *et al*. 2002). To quantify the GRF response magnitude, we measured the peak of this late response. To compare hand force between LG and FG, peak lateral hand forces were measured for FG and the time at which this occurred was used to measure the response magnitude for LG. Times of peak change in GRF and hand force (derivative) after stimulus onset were used as measures of response latency (Marsden *et al*. 2005). Body position and velocity were derived from the Fastrak head sensor. Peak lateral body position and velocity during GVS were used as measures of whole‐body movement magnitude.

### Experiment 2

After establishing that an active GVS‐evoked upper limb response only occurred during FG in Experiment 1 (see Results, Experiment 1), we then aimed to determine how the upper limb contributes to the direction of the whole‐body sway response. Head‐on‐body orientation was altered to determine how the craniocentric properties of GVS‐evoked postural responses would affect the upper limb response direction. The directions of the GRF and hand force vectors, as well as the whole‐body sway response, were calculated for each head posture.

Three targets (30 × 30 cm) were positioned ahead of the subject (70 cm). One target was aligned with the mid‐line (0°) of subjects and the other two were positioned 45° to the left and right. Subjects were instructed to orientate their head such that their nose was aligned to one of the targets (head forward, left or right). Two grip conditions were tested: NC and FG (same hand positions as Experiment 1). Once the head was positioned correctly, the subjects closed their eyes and the trial began. Two repeats for each of the six conditions were recorded: three head orientations (forward, left, right) × 2 grip conditions (NC and FG), providing a total of 12 trials.

In Experiment 1, subjects produced hand forces directed towards the anode electrode during FG (see Results, Experiment 1). Before analysing the direction of this active response in Experiment 2, it was first necessary to confirm its existence in each subject. We determined an upper limb response as being present if mediolateral (ML) force was directed towards the anode and exceeded 2 SD of baseline force (500 ms before GVS) for at least 250 ms. Three of twelve subjects did not meet this criterion and were removed from subsequent directional analysis.

##### Quiet standing body sway

Quiet standing was recorded for 15 s at the start of each trial without GVS. Whole‐body sway direction was determined by fitting a 95% confidence ellipse to body position data (Sparto & Redfern, 2001). Large (*a*) and small (*b*) ellipse vectors were measured. The angle between the largest ellipse vector and the anteroposterior (AP) axis was taken as the direction of sway, constrained between 0° and +180°. Ellipse eccentricity $\left(a/a^{2}\times b^{2}\right)$ was used as a measure of baseline sway asymmetry. If eccentricity is equal to 0 (i.e. a perfect circle), this would indicate that the ellipse was not skewed in any particular direction. As eccentricity becomes closer to 1 (i.e. a straight line), the ellipse becomes more skewed in a specific direction. Ellipse area (π *ab*) provided a measure of sway variability. The directions of GRF and hand force during quiet standing were determined in the same way as whole‐body sway. GRF and hand force were also summed before determining summed force baseline direction.

##### GVS response directions

Response directions were measured from the AP and ML components of the response at 0.4 s (GRF and hand forces) and 2 s (body position) post GVS onset (Mian & Day, 2014). Response direction was calculated as tan−1 ML / AP . Separately, we also summed the GRF and hand forces to measure the combined force vector direction.

### Statistical analysis

All data were analysed using Matlab (Mathworks Inc., Natick, MA, USA).

##### Linear data (Experiments 1 and 2)

Repeated‐measures ANOVA was used to test for main effects of conditions. To test for significant hand force responses in Experiment 1, one‐sample *t* tests were used to compare peak hand forces to zero. SPSS, version 19 (IBM Corp., Armonk, NY, USA) was used for statistical testing. *P* < 0.05 was considered statistically significant.

##### Directional data (Experiment 2)

Descriptive statistics specific to circular data (i.e. circular mean and angular deviation) (± AD) (Zar, 2010) were used to analyse angular direction of body sway during quiet standing and GVS response directions. The mean direction is only meaningful when the sample of angles is not a uniform circular distribution. Therefore, mean direction was only calculated after the Rayleigh test for uniformity rejected a uniform distribution (*P* < 0.05) (Zar, 2010). To determine the difference between more than two conditions (e.g. three head orientations), ideally, a repeated‐measures ANOVA designed for circular data would be used. However, to our knowledge, no such test exists. We therefore used the Moore's test for paired circular data (Moore, 1980), which is the equivalent of a paired samples *t* test used for linear data, to investigate differences in response direction between conditions. Means, ADs and the Rayleigh test for circular data were analysed using CircStat toolbox for Matlab (Berens, 2009).

## Results

### Experiment 1

There was no effect of stimulus polarity (anode‐right *vs*. left) on the magnitude of the GRF (*F*_1,9_ = 1.60, *P* = 0.23) or hand force response (*F*_1,9_ < 0.001, *P* = 1.00). Therefore, both polarities were combined after inverting anode‐left data.

#### GRF

**Figure 2:**
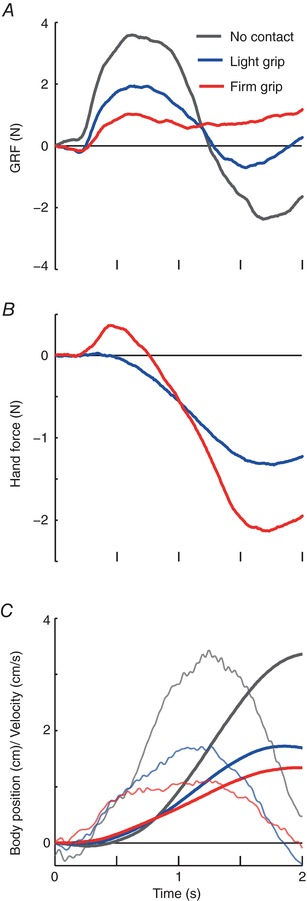
Representative GRF, hand force and body movement *A*, ML GRF during 2 s GVS (GVS onset is at 0 s) in the three grip conditions, for an individual subject. A positive force indicates one that would move the body towards the anode. *B*, ML hand force response during LG and FG. *C*, ML body position (thick traces) and velocity (thin traces). Positive body position/velocity indicates body movement towards the anode.

**Figure 3:**
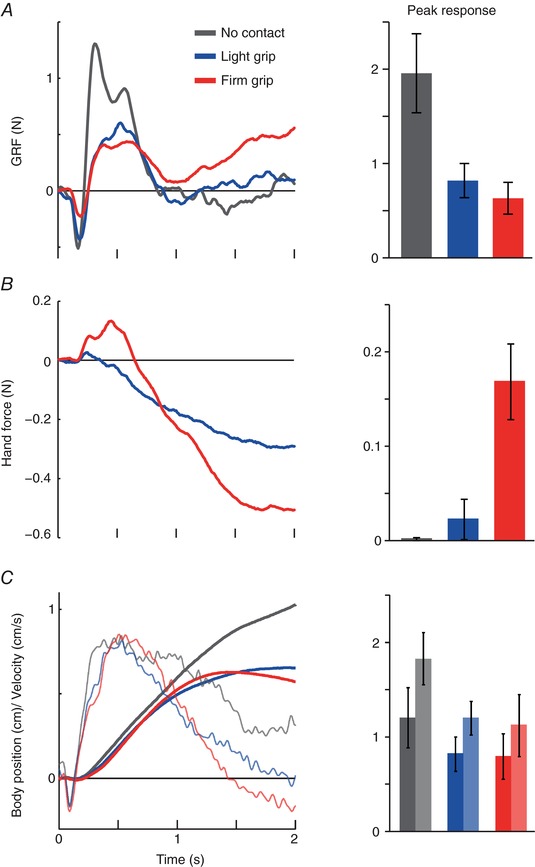
Mean GRF, hand force and body movement *A*, ML GRF response during GVS in the three grip conditions and corresponding peak (mean ± SE) GRF response towards the anode. There was a significant main effect of grip condition on peak GRF (*P* < 0.001). *B*, ML hand force response during LG and FG, and corresponding peak upper limb response. There was a significant main effect of grip condition on peak hand force (*P* = 0.001) and peak hand force was only significantly greater than zero in the FG condition (*P* = 0.002). *C*, ML body position (thick traces) and velocity (thin traces) and corresponding peak body position (dark bars) and velocity (light bars). There was a significant main effect of grip on peak body velocity (*P* = 0.01) but not position (*P* = 0.09).

Figure 2 shows mediolateral GRF, hand force and body sway responses to GVS for a representative subject. GVS evoked a GRF response directed towards the anode during no contact (NC), peaking at ∼600 ms (Fig. 2 *A*). This is consistent with the late component of the GRF response described previously. Analysis was focused on this component because it is responsible for generating the whole‐body movement (Marsden *et al*. 2002). Average responses are shown in Fig. 3. LG (mean ± SD grip force = 0.6 ± 0.5 N) caused a reduction in the peak GRF, which was further reduced during FG (peak GRF force NC: 1.97 ± 1.32 N; LG: 0.83 ± 0.56 N; FG: 0.64 ± 0.55 N) (Fig. 3 *A*), with a significant main effect of grip condition (*F*_2,18_ = 14.33, *P* < 0.001).

#### Hand force

Hand forces largely mirrored whole‐body movement during the LG condition (compare blue traces in Fig. 2 *B* and *C*). As the body swayed towards the anode electrode, this corresponded to a change in hand force tending to resist that motion. This suggests that the arm is acting similar to a passive spring. Although a tiny positive deflection can be seen on the mean trace (blue trace, Fig. 3 *B*), peak force was not significantly greater than zero (0.02 ± 0.07 N; *t*_9_ = 1.09, *P* = 0.30). By contrast, during FG (red trace; Figs 2 *B* and 3 *B*), the upper limb initially generated a significant force impulse directed towards the anode (0.17 ± 0.13 N; *t*_9_ = 4.18, *P* = 0.002). This early response was in the same direction as the GRF (red trace; Figs 2 *A* and 3 *A*), corresponding to an impulse that actively pushes the body towards the anode electrode. The differences in the hand force response between grips were confirmed by a significant main effect of grip condition on peak hand force (*F*_2,18_ = 10.68, *P* = 0.001). The onset latency was 256 ± 84 ms, which was not significantly different from the GRF latency (267 ± 45 ms; *t*_9_ = 0.36, *P* = 0.73).

#### Whole‐body sway

GVS also evoked a whole‐body movement that was directed towards the anode electrode for all conditions (Figs 2 *C* and 3 *C*). Body velocity responses became smaller during LG compared to NC and smaller again for the FG condition (NC: 1.8 ± 0.9 cm s^−1^; LG: 1.2 ± 0.6 cm s^−1^; FG: 1.1 ± 1.1 cm s^−1^) (Fig. 3 *C*), with a significant main effect of grip condition (*F*_2,18_ = 5.82, *P* = 0.01). Although the same trend can be observed for body position, this did not reach significance (NC: 1.2 ± 1.0 cm; LG: 0.8 ± 0.6 cm; FG: 0.8 ± 0.8 cm; *F*_2,18_ = 2.83, *P* = 0.09).

### Experiment 2

In Experiment 1, the upper limb produced an active response to GVS only when firmly grasping the support. In Experiment 2, we investigated the directional nature of this response under three different head orientations (+45, 0, −45°). Three of the twelve subjects demonstrated no significant GVS‐evoked increase in hand force above baseline and so were excluded from this analysis (for response criteria, see Methods).

#### Head orientation

There was no significant effect of grip condition (NC *vs*. FG) upon head orientation (Moore's test; *R* ^*^_9_ ≤ 0.70, *P* > 0.05). As expected, head yaw angle was significantly different between head orientation conditions (*R* ^*^_9_ ≥ 2.21, *P* < 0.05). Mean ± AD head yaw angles were: head forward, 1 ± 5°; head left, −40 ± 9°l and head right, 36 ± 13°.

#### Baseline forces and body sway

Previous research has shown that the direction of GVS‐evoked sway is biased towards the axis of instability when finger contact causes baseline sway to be more stable in one particular axis (Mian & Day, 2014). We therefore analysed baseline body sway and forces to determine whether FG produced such anisotropic effects.

**Figure 4:**
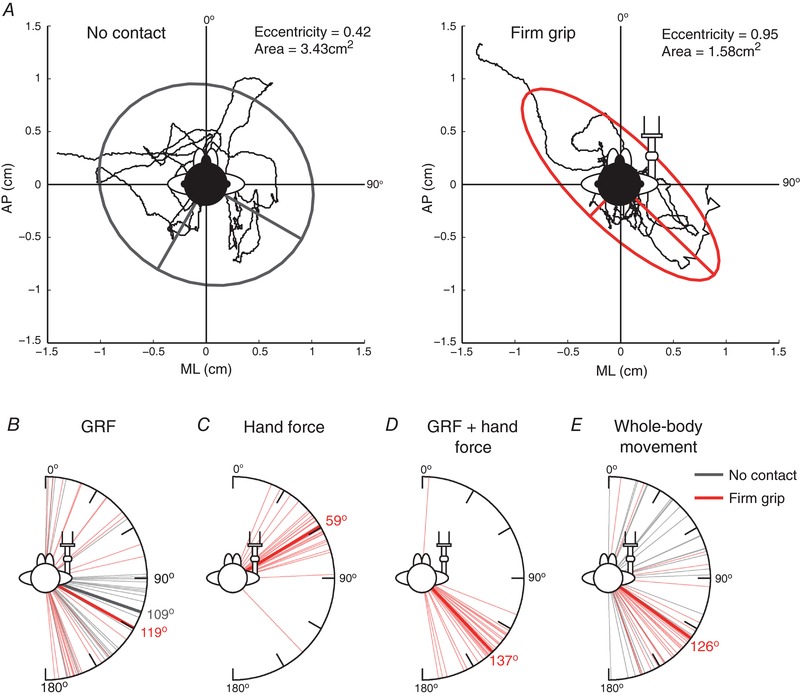
Force and body sway directions during quiet standing *A*, an example of a 95% confidence ellipse fitted to a representative body sway (derived from motion capture sensor fixed to the head of a subject) in ML and AP axis during 15 s of quiet standing with head forward (0°) during NC and FG, respectively. Large and small ellipse vectors are shown. Baseline directions were measured as the angle between the large ellipse vector and the AP axis. *B*–*E*, baseline vectors for all subjects during quiet standing (thin lines) (note head orientation conditions are not separated) for GRF (*B*), hand force (*C*), GRF and hand force summed (*D*) and whole‐body movement (*E*). Mean force/position vectors (thick continuous lines) are only shown for conditions were the vectors were non‐uniformly distributed as determined by a Rayleigh test.

An example of how baseline directions were measured is shown in Fig. 4 *A*. To determine the direction of baseline forces and body position, ellipses were fitted to 15 s of data during NC and FG before any GVS was delivered. The angle of the ellipse vector was then used as a measure of baseline direction. Ellipse eccentricity was used as a measure of the strength of the ellipse direction and ellipse area as a measure of variability. To compare baseline directions between grip conditions (NC *vs*. FG), head orientations (forward, left, right) were combined within grip conditions.

Baseline force and body sway vectors during quiet standing are shown in Fig. 4 *B*–*E*. Baseline GRF vectors (Fig. 4 *B*) were non‐uniformly distributed in both grip conditions (Rayleigh test; *P* ≤ 0.016), with mean ± AD vector direction of 109 ± 45° and 119 ± 58° in the NC and FG condition, respectively. However, these GRF vectors were not significantly different (*R* ^*^_27_ = 0.78, *P* > 0.05). Ellipse eccentricity was significantly reduced in the NC condition compared to FG (NC: 0.64 ± 0.1, FG: 0.75 ± 0.1; *t*_26_ = 4.21, *P* < 0.001). Therefore, although the GRF baseline force vectors were significantly directed during NC, the strength of this directedness was less than the FG condition. There was also a significant effect of grip condition on baseline GRF variability (ellipse area), with reduced variability during FG compared to NC (NC: 32.8 ± 15.0N^2^, FG: 13.7 ± 8.3N^2^; *t*_26_ = 6.10, *P* < 0.001).

During FG, the baseline hand force vector (Fig. 4 *C*) was significantly directed towards 59 ± 19° (Rayleigh test; *P* < 0.001, eccentricity = 0.85 ± 0.1, area = 9.9 ± 9.4 N^2^), approximately aligned with the position of the handle (∼45°). When GRF and hand forces were summed (Fig. 4 *D*), the force vector was significantly directed at 137 ± 25° (*P* < 0.001, eccentricity = 0.74 ± 0.1, area = 12.2 ± 6.5 N^2^), approximately orthogonal to the handle position.

The whole‐body sway direction (Fig. 4 *E*) reflects the summed GRF and hand force vectors during FG, with body sway significantly directed towards a mean angle of 126 ± 33° (*P* < 0.001). By contrast, during NC, baseline body sway was uniformly distributed in all directions (*P* = 0.29). Ellipse eccentricity was significantly larger during FG compared to NC (NC: 0.77 ± 0.1, FG: 0.86 ± 0.1; *t*_26_ = 3.94, *P* = 0.001) and ellipse area was significantly smaller during FG (NC: 11.7 ± 6.3 cm^2^, FG: 3.7 ± 2.7 cm^2^; *t*_26_ = 6.86, *P* < 0.001). Hence, FG did produce anisotropic effects upon baseline body sway that we take into account when considering the GVS‐evoked response direction below.

#### GVS responses during no contact

**Figure 5:**
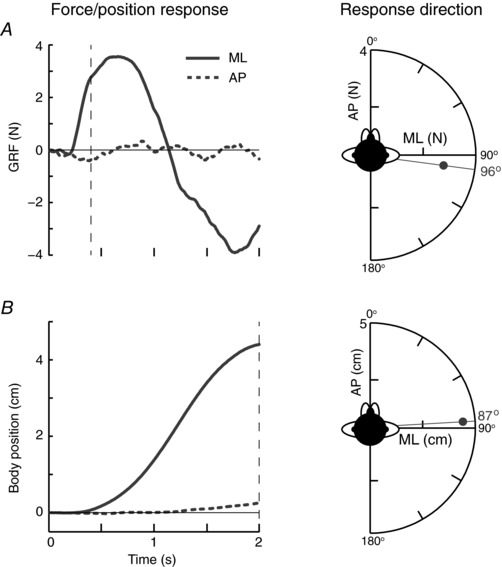
Representative response with the head forward during no contact GRF (*A*) and body position (*B*) in the AP (dashed trace) and ML (solid trace) axis during GVS. AP and ML force and body position responses were measured at 0.4 and 2 s, respectively (vertical dashed lines) and plotted against each other (grey dot, response direction). These values also indicate the magnitude of the response.

Figure 5 summarizes the GRF (Fig. 5 *A*) and body position (Fig. 5 *B*) response to GVS in the NC condition with the head forward for a representative subject. The main GRF response was in the ML direction. This consisted of an initial slight dip, followed by a much larger positive deflection in ML force. These two components constitute the short and medium‐latency response to GVS, with the latter being responsible for the evoked body sway (Marsden *et al*. 2002). The direction of the force vector was calculated from the AP and ML traces at 0.4 s. This resulted in a response direction of 96°, which is approximately aligned with the inter‐aural axis (∼90°) of subjects. The body sway vector (measured at 2 s) reflected the force, being directed towards the anode at 87° (Fig. 5 *B*).

**Figure 6:**
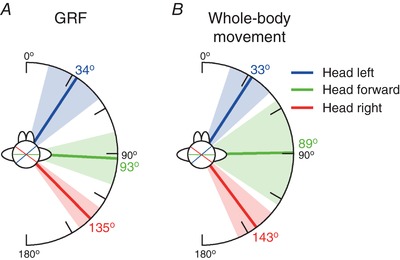
Mean response directions for different head orientations during no contact Mean GRF (*A*) and whole‐body movement (*B*) response directions during GVS with the head orientated to the left (blue), forward (green) and right (red). Shaded areas indicate ± AD. Axes shown on the head indicate the line of the inter‐aural axis (orthogonal to head angle) showing the head angle.

Mean response directions for the three head orientation conditions are shown in Fig. 6. All GVS responses were significantly directional, as determined by a Rayleigh test (*P* ≤ 0.001). With the head facing forward, mean ± AD GRF response direction was 93 ± 17°, being aligned with the inter‐aural axis (91°). Whole‐body movement reflected the GRF response, and was directed at 89 ± 34°. Turning the head left or right caused the GRF vector to be significantly rotated by a similar amount (left: 34 ± 19°, *R* ^*^_9_ = 1.61, *P* < 0.05; right: 135 ± 9°, *R* ^*^_9_ = 1.65, *P* < 0.05). This was the same for whole‐body movement direction (left: 33 ± 14°, *R* ^*^_9_ = 1.50, *P* < 0.05; right: 143 ± 13°, *R* ^*^_9_ = 1.57, *P* < 0.05). Hence, during the NC condition, the GVS response behaved in a craniocentric fashion, staying fixed in head co‐ordinates.

#### GVS response during FG

**Figure 7:**
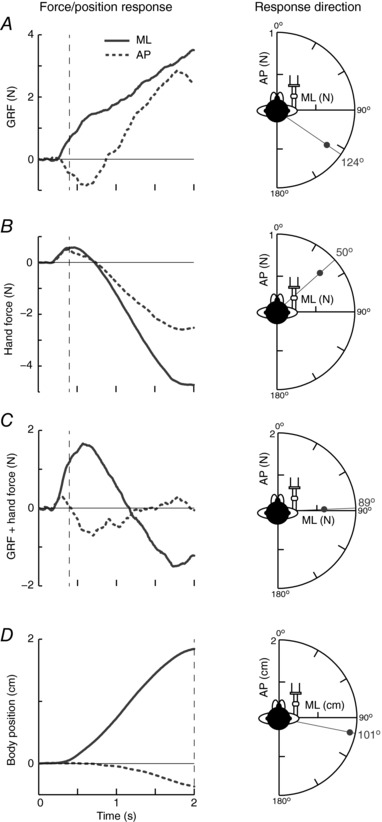
Representative response with the head forward during FG GRF (*A*), hand force (*B*), GRF and hand forces summed (*C*) and body position (*D*) response in the ML (solid trace) and AP (dashed trace) axis during GVS. Response directions were derived as described in Fig. 5.

Figure 7 displays a representative response to GVS from a subject engaging in FG with their head forward. The GRF response was directed backward (AP) and towards the anode (ML), with an angle of 124° (Fig. 7 *A*). This is clearly no longer aligned with the inter‐aural axis. By contrast, the hand generated force not only towards the anode, but also forward (50°) (Fig. 7 *B*). When the GRF and hand forces were summed together, the direction of the overall force vector was 89° (Fig. 7 *C*). This was similar to the direction of whole‐body movement (101°) (Fig. 7 *D*). The overall force and sway response was therefore aligned approximately with the inter‐aural axis, as seen during the NC condition (Fig. 6).

**Figure 8:**
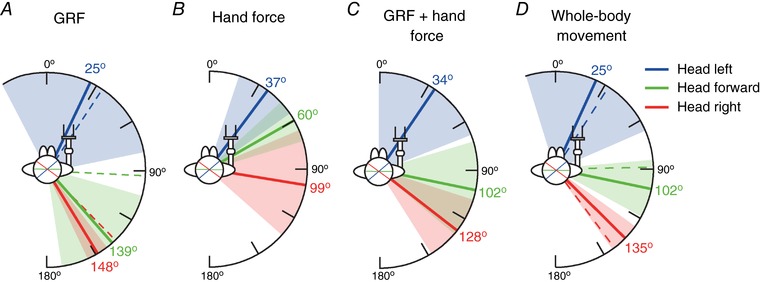
Mean response directions for different head orientations during FG Direction of GVS‐evoked responses for GRF (*A*), hand force (*B*), GRF and hand forces summed (*C*) and whole‐body movement (*D*) for the three head orientations during FG (thick lines). Shaded areas indicate ± AD. Response directions during NC (dashed lines) are shown for comparison between grip conditions. Note, for hand force (*B*) and GRF + hand force (*C*), only the FG condition is shown because no hand response was recorded in the NC condition. Axes shown on the head indicate the line of the inter‐aural axis showing the head angle.

Mean GVS response directions for the three head orientations during FG are shown in Fig. 8. All responses were significantly directional (*P* ≤ 0.048). With the head forward, the mean ± AD GRF vector was 139 ± 33° (Fig. 8 *A*). Compared to the NC condition, this was significantly rotated by 46° clockwise (*R* ^*^_9_ = 1.45, *P* < 0.05) and was aligned towards the direction of baseline summed forces (GRF+hand force = 137°) (Fig. 4 *D*). With the head left or right, the difference in GRF response direction between the FG and NC condition was smaller and only significant when facing to the right (left: 25 ± 53°, *R* ^*^_9_ = 0.79, *P* > 0.05; right: 148 ± 6°, *R* ^*^_9_ = 1.51, *P* < 0.05).

With the head forward, the hand force vector was 60 ± 13° (Fig. 8 *B*). This was approximately orthogonal (−79°) to the GRF vector. Turning the head left or right significantly altered the upper limb response direction, causing it to become aligned towards the inter‐aural axis (left: 37 ± 18°, *R* ^*^_9 _= 1.21, *P* < 0.05; right: 99 ± 32°, *R* ^*^_9_ = 1.44, *P* < 0.05).

As seen in the representative subject, summing the GRF and hand forces caused the combined vector to become aligned closer towards the inter‐aural axis (Fig. 8 *C*). With the head forward, the summed force direction was 102 ± 28°. When the head was turned to left or right, the summed force vector was significantly altered (compared to the head forward condition) towards the inter‐aural axis (left: 34 ± 34°, *R* ^*^_9_ = 1.35, *P* < 0.05; right: 128 ± 20°, *R* ^*^_9_ = 1.21, *P* < 0.05).

Although the direction of the GRF response was skewed with the head forward during FG compared to NC, the direction of whole‐body movement was unaffected (Fig. 8 *D*). With the head forward, body movement was directed at 102 ± 17°, reflecting the summed GRF and hand force vector. This was not significantly different to the direction of body movement seen in the NC condition (*R* ^*^_9_ = 0.89, *P* > 0.05). This was also the case when the head was orientated to the left (25 ± 42°, *R* ^*^_9_ = 0.74, *P* > 0.05). However, as shown for GRF, sway direction was slightly but significantly altered by grip when facing to the right (135 ± 9°, *R* ^*^_9_ = 1.58, *P* < 0.05).

## Discussion

With the exception of Britton *et al*. (1993), previous demonstrations of vestibular influence on the upper limb have been restricted mainly to the study of reaching movements, when the arm is not actively engaged in balance (Bresciani *et al*. 2002; Mars *et al*. 2003; Blouin *et al*. 2015; Smith & Reynolds, 2017). In the present study, we applied GVS to subjects who were standing normally when holding onto a fixed object. We observed stimulus‐related forces generated by the upper limb. These forces were systematically altered by grip type and head orientation, and were co‐ordinated with GRF to move the body in a direction intended to compensate for the vestibular perturbation.

The present study aimed to answer three questions. First, does the magnitude and direction of the GVS‐evoked upper limb force depend upon grip context? We found that changes in hand grip altered the upper limb response both qualitatively and quantitatively. The LG condition involved a very light finger and thumb grip, with pinch force within 1 N. Such levels of force can provide abundant sensory information with minimal mechanical stabilization (Holden *et al*. 1994). In this situation, GVS evoked a relatively slow, continuous and unidirectional build‐up of lateral hand force for the duration of the stimulus (blue trace, Fig. 3 *B*). This force was directed towards the cathodal ear (acting on the body). Given that GVS evokes sway towards the anodal ear, this upper limb force would act to resist the whole‐body response to the vestibular perturbation. Therefore, during LG, the arm did not drive the GVS sway response but instead reflected it. In other words, the arm appeared to behave similar to a passive spring, simply registering cutaneous forces as a result of body motion. Such cutaneous input could provide additional balance‐related sensory information that would conflict with that of GVS (Day *et al*. 2002). This would act to limit the sway response to GVS and may explain why the sway response was smaller during LG compared to the NC condition, as also shown by Britton *et al*. (1993). During FG, subjects used their whole hand to firmly grip a ball and handle. This changed the nature of the upper limb response, with the appearance of an early force impulse in the opposite direction to that of LG (red trace, Fig. 3 *B*). This impulse is the same direction as the GRF, acting to drive the body towards the anodal ear. Hence, a simple change in grip is sufficient to convert the arm from being a passive responder to being an active generator of body movement. However, 25% of subjects did not generate this impulse (Experiment 2), precluding calculation of a response direction. Although we did not measure grip force during the FG condition, it may be that these subjects did not grip sufficiently strongly to engage the hand in balance. Subsequent to the early impulse, the force reversed direction and began to resemble the pattern observed during LG, albeit larger. The absence of the early force impulse during LG could simply be the result of a lack of strength associated with that particular grip. Overall, peak hand forces produced during FG were approximately double those of LG (−0.25 N *vs*. −0.5 N) (Fig. 3 *B*). However, the early active force impulse observed during FG was only ∼ 0.1 N, suggesting that strength limitations were not a factor in its absence during LG. Instead, the change in grip context is a cue for the nervous system to transform the arm from a passive listener to an active participant in the balance process.

The second question concerned the direction of the GVS‐evoked hand force vector and whether it is systematically altered by head orientation in a craniocentric fashion. To answer this, we focussed on the early force impulse seen during FG and observed the effect of head yaw upon this active response. However, to confirm previous findings, we started by measuring the GRF vector in the absence of hand contact. With the head forward, this vector was oriented orthogonally to head direction (93°). Turning the head to the left or right caused the GRF vector to rotate by a very similar amount, which is consistent with the craniocentric principle (Lund & Broberg, 1983; Pastor *et al*. 1993; Mian & Day, 2009) (Fig. 6 *A*). Next, we measured the direction of the hand force vector during the FG condition. As for the GRF vector, this was significantly affected by head orientation, although the relationship was not systematic. In particular, the head‐forward and head‐right vectors were skewed in a counter‐clockwise direction (Fig. 8 *B*). To understand the cause and consequences of this skew, we must consider the direction of the simultaneous GRF vectors, which brings us to our third question: how well is upper limb force integrated with the GRF vector, and how does this affect whole‐body sway?

FG significantly skewed the GRF vector. This is most apparent during the head‐forward condition, where it is oriented at 139° *vs*. 93° during NC (Fig. 8 *A*). Recent research has described a similar violation of craniocentricity when baseline sway becomes more stable in one axis (i.e. anisotropic) (Mian & Day, 2014). To determine whether this was the case, we compared baseline forces and body sway between conditions. Although baseline GRF directions were similar between conditions, whole‐body sway became preferentially destabilized towards a 126° axis during FG compared to no skew during NC (Fig. 4 *E*). The anisotropic effect of FG on body sway reflected the baseline summed GRF and hand force vector, which was directed towards 137° (Fig. 4 *D*). This would explain why the GVS response was biased towards that direction during the head‐forward condition. In comparison, minimal skew was observed with the head right or left, presumably because the evoked sway direction was either aligned with (or orthogonal to) the axis of instability, respectively. Hence, FG appeared to cause a large deviation in the GRF vector only during the head‐forward condition, as a result of changes in baseline sway. To discover the consequences of these deviations for the overall response to GVS, we summed the GRF and hand force and computed the resulting vector. The summed vectors bear a stronger resemblance to the GRF vector during NC. This suggests that the skewed deviations observed in the upper and lower limbs cancel each other to some extent. The ultimate effect of such a cancellation process would be to preserve the direction of body sway. Indeed, with the head forward the GVS sway response was similarly craniocentric for both the NC and FG conditions, with a difference of only 13° (Fig. 8 *D*, green traces), compared to 46° for the GRF response (Fig. 8 *A*, green traces). When the head was turned to left or right, there were only small deviations in body sway directions during FG, as seen in the GRF response. One potential limitation is our use of a motion capture sensor fixed to the head to derive whole‐body movement. However, GVS has been shown to produce very similar sway responses when measured either at the head or trunk (Day *et al*. 1997).

Figure 8 *D* clearly shows that the GVS sway response was similarly craniocentric for both the NC and FG conditions. Such cancellation was not apparent in the findings of Mian and Day (2014), who examined the GVS‐evoked summed force response during light touch. However, our observations during LG show that the arm does not generate active forces in response to GVS during such low‐force contact. This suggests that the cancellation of skewed forces between hand and foot only occurs if the hand is an active participant in driving the response to the vestibular perturbation. Under these circumstances, the principle of craniocentricity is preserved.

In summary, we have demonstrated vestibular‐evoked forces in the upper limb that are designed to counteract a false sense of body motion. Under conditions of LG, the observed hand forces did not cause the body sway response but were consequential to it. For the hand to generate forces that drive the body sway response to GVS required a sufficient FG. Under these conditions, the hand forces were co‐ordinated with the ground reaction forces to move the body in the same direction as seen when the upper limb was not engaged in balance.

## Additional information

### Competing interests

The authors declare that they have no competing financial interests.

### Author contributions

This study was performed at the School of Sport, Exercise and Rehabilitation sciences, University of Birmingham, Birmingham, UK. All of the authors contributed to the conception and design of the experiments. CPS collected and assembled data. CPS and RFR contributed to the analysis and interpretation of data and drafting the article. All authors revised the manuscript for important intellectual content and approved the final version submitted for publication.

### Funding

This work was supported by the BBSRC (BB/M027880/1 and BB/100579X/1), the European Commission FP7 CoDyCo project (no. 600716) and a pump‐priming grant from the Centre for Computational Neuroscience and Cognitive Robotics at the University of Birmingham. CPS is supported by the BBSRC Midlands Integrative Biosciences Training Partnership doctoral programme.
